# Obstructive sleep apnea is linked to inflammatory changes and motor impairment in Parkinson’s disease

**DOI:** 10.3389/fimmu.2026.1808550

**Published:** 2026-05-18

**Authors:** Carlos Mauricio Oliveira de Almeida, Marcello Facundo do Valle-Filho, Raissa Costa Said, Jefferson Macedo Dantas, Agatha Lopes Azedo, Mariana Carvalho Ferreira, Emillaine Noronha, Yenly Gonzalez Perez, Manoel Alves Sobreira-Neto, Antônio Luiz Ribeiro Boechat Lopes, Marcus Vinicius Della Coletta, Nani Oliveira Carvalho, Andréa Teixeira-Carvalho, Diego Monteiro Carvalho, Gemilson Soares Pontes

**Affiliations:** 1 State University of Amazonas (UEA), School of Health Sciences, Manaus, Brazil; 2 State University of Amazonas, School of Health Sciences, Researcher in the Sleep Disorders Sector at Hospital Adriano Jorge, Manaus, Brazil; 3 Federal University of Amazonas (UFAM), Manaus, Brazil; 4 Federal University of Ceará (UFC), Fortaleza, Brazil; 5 Instituto René Rachou – Fundação Oswaldo Cruz (FIOCRUZ)-Minas, Belo Horizonte, Brazil; 6 Laboratory of Virology and Immunology, National Institute of Amazonian Research (INPA), Manaus, Brazil

**Keywords:** immune profile, neuroinflammation, obstructive sleep apnea, Parkinson’s disease, sleep fragmentation

## Abstract

**Introduction:**

Obstructive sleep apnea (OSA) is prevalent among patients with Parkinson’s disease (PD), yet its contribution to neuroinflammation and disease progression remains poorly understood. This study investigated the association between OSA severity, sleep parameters, and systemic cytokine/chemokine profiles in PD.

**Methods:**

In this cross-sectional study, 48 patients underwent clinical evaluation, polysomnography (PSG), and multiplex cytokine profiling. Participants were classified as PD+OSA^+^ or PD+OSA^-^ based on PSG, with additional stratification by OSA severity. Multivariate and network analyzes were applied to identify inflammatory signatures.

**Results:**

OSA was diagnosed in 54.2% of PD patients and was associated with older age, poorer quality of life, and higher UPDRS-II/III scores (p < 0.05). The PD+OSA^+^ group exhibited reduced REM sleep, increased arousals, and higher oxygen desaturation indices. Cytokine analysis revealed that moderate-to-severe OSA was associated with a mixed Th1/Th2 inflammatory axis involving IL-6, TNF-α, IFN-γ, IL-13, CCL2, CXCL1, and IL-4, while mild OSA was associated with innate activation and chemotaxis (IL-1β, IL-18, CXCL10, GM-CSF). Network modeling demonstrated dense cytokine connectivity in PD+OSA^+^, suggesting systemic immune reorganization driven by intermittent hypoxia.

**Discussion:**

OSA is a prevalent disorder in PD, affecting over half of the patients, and is linked to sleep fragmentation and a proinflammatory environment driven by Th1/Th2 and chemokine signaling, which may contribute to worsening motor and non-motor symptoms.

## 1 Introduction

Parkinson’s disease (PD) is the second most common neurodegenerative disorder in the elderly, affecting about 1% of individuals over 60 years of age (1, 2). Beyond the characteristic dopaminergic neuronal loss, growing evidence suggests that neuroinflammatory mechanisms significantly contribute to PD pathogenesis and symptom progression (3, 4). Moreover, comorbid conditions such as obstructive sleep apnea (OSA) may exacerbate neuroinflammatory stress and neuronal vulnerability, underscoring the complex interplay between systemic factors and neurodegeneration in PD (5).

Sleep disorders (SDs) are among the most prominent non-motor symptoms (NMS) of PD and may precede motor manifestations by years or even decades (6, 7). They affect up to 90% of individuals with PD, with OSA, insomnia, and rapid eye movement (REM) sleep behavior disorder (RBD) being the most prevalent (8). OSA, characterized by recurrent upper airway obstruction leading to intermittent hypoxemia, sleep fragmentation, and excessive daytime sleepiness, is ubiquitous—affecting 9–38% of the general population. However, in PD, prevalence estimates vary substantially (20–70%), likely owing to differences in study design, diagnostic criteria and population characteristics (9–11). These sources of heterogeneity warrant caution when comparing prevalence across studies.

The relationship between OSA and PD appears to be bidirectional, underpinned by overlapping neurophysiological and inflammatory mechanisms (12–14). Neurodegeneration in PD may compromise upper airway motor control and respiratory regulation, increasing the risk of OSA. Conversely, OSA-related chronic intermittent hypoxia enhances oxidative stress and triggers neuroinflammatory cascades, promoting glial activation, blood–brain barrier dysfunction, and neuronal injury (15). These inflammatory processes are believed to accelerate neurodegeneration and α-synuclein pathology, thereby linking sleep-disordered breathing to disease progression (16). Despite these mechanistic insights, the extent to which OSA amplifies PD’s clinical expression through inflammatory pathways remains insufficiently explored.

Few studies have conducted integrative analyzes of how OSA modulates both motor and non-motor phenotypes in PD and how this interaction relates to systemic inflammation. Evidence suggests that OSA-related intermittent hypoxia and sleep fragmentation amplify oxidative stress, trigger microglial activation, and sustain low-grade systemic inflammation, thereby accelerating nigrostriatal neurodegeneration and worsening motor and cognitive dysfunction in PD (12, 17, 18).

Recent findings have identified significant alterations in pro-inflammatory cytokines, such as IL-6 and TNF-α, and reduced serum BDNF levels in PD patients with comorbid OSA, supporting the concept of a shared inflammatory environment underlying both disorders (5, 19). Furthermore, laryngopharyngeal motor dysfunction in PD may predispose patients to upper airway collapse during sleep, reinforcing the bidirectional relationship between neuromuscular impairment and sleep-disordered breathing (20). In this context, the present study aimed to determine the frequency of OSA among PD patients and to explore its associations with quality of life, clinical and polysomnographic parameters, and inflammatory cytokine profiles, to clarify the potential role of the inflammation–sleep–neurodegeneration axis in PD pathophysiology.

## 2 Methods

### 2.1 Subjects and clinical assessments

This observational cross-sectional study was conducted at the Observatory Center for Otolaryngological Diseases, which is part of the Otolaryngology Department, and at the Sleep Clinic of the Adriano Jorge Hospital Foundation (FHAJ) in Manaus, Brazil. The study was approved by the local Research Ethics Committee under No. 58684622.2.0000.0007 and was conducted from August 2023 through March 2025. All participants provided written informed consent.

Eligible patients underwent a two-step evaluation process. The first step consisted of neurological assessment by a specialist in movement disorders and sleep medicine, in addition to otolaryngological evaluation and flexible nasofibroscopy. A semi-structured questionnaire was used to collect data on disease duration, clinical manifestations, sleep-related information, medications, and associated conditions. The second step involved a full overnight type 1 polysomnography (PSG) at the FHAJ. All patients were assessed during the “on” phase of their medication cycle.

Adults with PD (>18 years) diagnosed according to the UK Parkinson’s Disease Society Brain Bank criteria and Hoehn and Yahr stages 1 to 3 (21, 22).

The exclusion criteria were as follows:

1. Dementia.
2. Active neoplastic disease.
3. Severe or decompensated systemic diseases.
4. Alcohol abuse or illicit drug use.
5. Language (aphasia, dysphasia) or speech (dysarthria, hypophonia) disorders preventing questionnaire comprehension.
6. Prior surgical treatment for OSA or PD.
7. Atypical Parkinsonism.
8. Current treatment with Continuous Positive Air Pressure (CPAP) for OSA.

### 2.2 Clinical and sleep questionnaires

The following validated instruments were used: Pittsburgh Sleep Quality Index (PSQI), Epworth Sleepiness Scale (ESS), Parkinson’s Disease Sleep Scale (PDSS-1), REM Sleep Behavior Disorder Screening Questionnaire (RBDSQ-BR), Hoehn and Yahr staging scale, Unified Parkinson’s Disease Rating Scale (UPDRS parts 1–4), the STOP-BANG questionnaire for OSA screening (22–29), and the Mini-Mental State Examination (MMSE) (30).

Restless Legs Syndrome (RLS) was diagnosed through a face-to-face clinical interview based on criteria from the International Classification of Sleep Disorders – 3rd revision (ICSD-3R) (31) The diagnosis of OSA was established according to the ICSD-3R criteria (32).

For analytical purposes, a PSQI score >5 was considered indicative of poor sleep quality, and an ESS score >10 indicated excessive daytime sleepiness (EDS). STOP-BANG scores were categorized as low risk (0–2), intermediate risk (3–4), and high risk (5–8) for OSA. For the RBDSQ-BR, a score >5 was considered suggestive of probable RBD.

### 2.3 Polysomnography and nasovideolaryngoscopy

PSG was performed using a video-synchronized system with a 45-channel digital polygraph (The Neurovirtual BW-3 Plus Neurovirtual Ltda, Brazil). The record montage followed standard specifications and included a six-channel EEG (10–20 system) with electrodes at F3-A2, F4-A1, C3-A2, C4-A1, O2-A1, and O1-A2; bilateral electrooculography (EOG); a single ECG channel; and three EMG channels to assess muscle activity in the mentalis, anterior tibialis, and superficial flexor muscles of the upper limbs. Additional sensors included an oral pressure cannula, a nasal thermistor, thoracic and abdominal piezoelectric belts, a body position sensor, an integrated pulse oximeter. All recordings were synchronized with an infrared video system to allow behavioral analysis (Intelbras, Brazil),All sensors were manufactured by Neurovirtual Ltda, Brazil.

Sleep staging was performed according to the criteria of the American Academy of Sleep Medicine (AASM) by trained experts blinded to the patients’ clinical complaints (33).Flexible nasovideolaryngoscopy was performed using the Ultra Slim 3.4 mm scope (Heine-Sass Wolf GmbH) by an otolaryngologist. The images were stored on a computer and reanalyzed for classification according to the Müller maneuver.

### 2.4 Plasma cytokine/chemokine profiling

To investigate whether comorbid OSA is associated with broad alterations in peripheral immune organization in Parkinson’s disease, we adopted a hypothesis-driven targeted multiplex immune profiling approach. Peripheral blood (5 mL) was collected in EDTA tubes from patients diagnosed with Parkinson’s disease, either with or without comorbid OSA. Samples were centrifuged at 1,500 g for 10 minutes to isolate plasma, which was then aliquoted and stored at −80 °C until analysis. Plasma levels of cytokines and chemokines were quantified using the Invitrogen™ ProcartaPlex™ Human Th1/Th2 Cytokine and Chemokine Panel 1, 20-plex (Thermo Fisher Scientific), following the manufacturer’s instructions. The panel included Th1/Th2 cytokines — GM-CSF, IFN-γ, IL-1β, IL-2, IL-4, IL-5, IL-6, IL-8, IL-12p70, IL-13, IL-18, and TNF-α — and chemokines: Eotaxin (CCL11), GRO-α (CXCL1), IP-10 (CXCL10), MCP-1 (CCL2), MIP-1α (CCL3), MIP-1β (CCL4), RANTES (CCL5), and SDF-1α. Measurements were performed using a Luminex^®^-based platform, and analyte concentrations were determined through a five-parameter logistic regression curve, as implemented in the accompanying analysis software.

### 2.5 Statistical analysis

Numerical variables were summarized as means and standard deviations, while categorical variables were presented as frequencies and percentages. Group comparisons were performed using Student’s t-test for normally distributed data and the Mann–Whitney U test for non-normally distributed data. Categorical variables were compared using the chi-square (χ²) test or Fisher’s exact test as appropriate. For the STOP-BANG variable, the Kruskal–Wallis test was used. A significance level of *p* ≤ 0.05 was adopted. For multivariate analysis, binary logistic regression was performed to identify predictors of OSA. The Variance Inflation Factor (VIF) was used to assess multicollinearity among numerical predictor variables, and those with VIF > 3 (e.g., AHI and RDI) were excluded. Independent variables with *p* < 0.05 in univariate analysis (age, UPDRS-3, UPDRS-2, total sleep time, desaturation index, minimum O_2_ saturation, snore index, arousal index, and PLMi) were included in the initial model, and variables with the highest *p*-values were progressively removed to reach the final adjusted model.

Plasma levels of cytokines from Parkinson’s disease patients with and without obstructive sleep apnea (PD+OSA+ vs PD+OSA−) were analyzed through multivariate and network-based approaches, with additional stratification of PD+OSA+ cases by OSA severity (mild vs moderate/severe). Raw data were imported using the readxl and data.table packages and preprocessed in RStudio (version 2025.05.1 Build 513). All cytokine values were standardized by z-score transformation (mean = 0, SD = 1) prior to analysis. Univariate comparisons between groups were performed using Welch’s t-test, and adjusted group comparisons were additionally assessed using analysis of covariance (ANCOVA) with sex and age as covariates. A two-tailed p-value < 0.05 was considered statistically significant. Heatmaps were constructed using the ComplexHeatmap and circlize packages in R, based on Euclidean distance and Ward.D2 clustering method, with annotation of experimental groups and expression clusters.

Pairwise correlations between cytokines were calculated using Spearman’s method (rcorr function from Hmisc package), and strong correlations were defined as ρ ≥ 0.60 for positive and ρ ≤ –0.60 for negative correlations (p < 0.05). Correlation matrices were visualized using OriginPro 2025, and functional correlation networks were built using Cytoscape (version 3.10.1). Network edges were weighted by absolute correlation values (|ρ|), and node strength was computed as the sum of incident edge weights. Community detection was performed using the Louvain algorithm for modularity optimization. Between-group changes in network topology were illustrated using Sankey plots.

Principal Component Analysis (PCA) and Linear Discriminant Analysis (LDA, MASS package) were applied to z-scores to evaluate group separation. The first latent variable (LV1) and LD1 scores were used to assess discriminatory performance with leave-one-out cross-validation (LOOCV) and area under the curve (AUC) metrics.

## 3 Results

### 3.1 Characteristics of the participants

**Figure 1:**
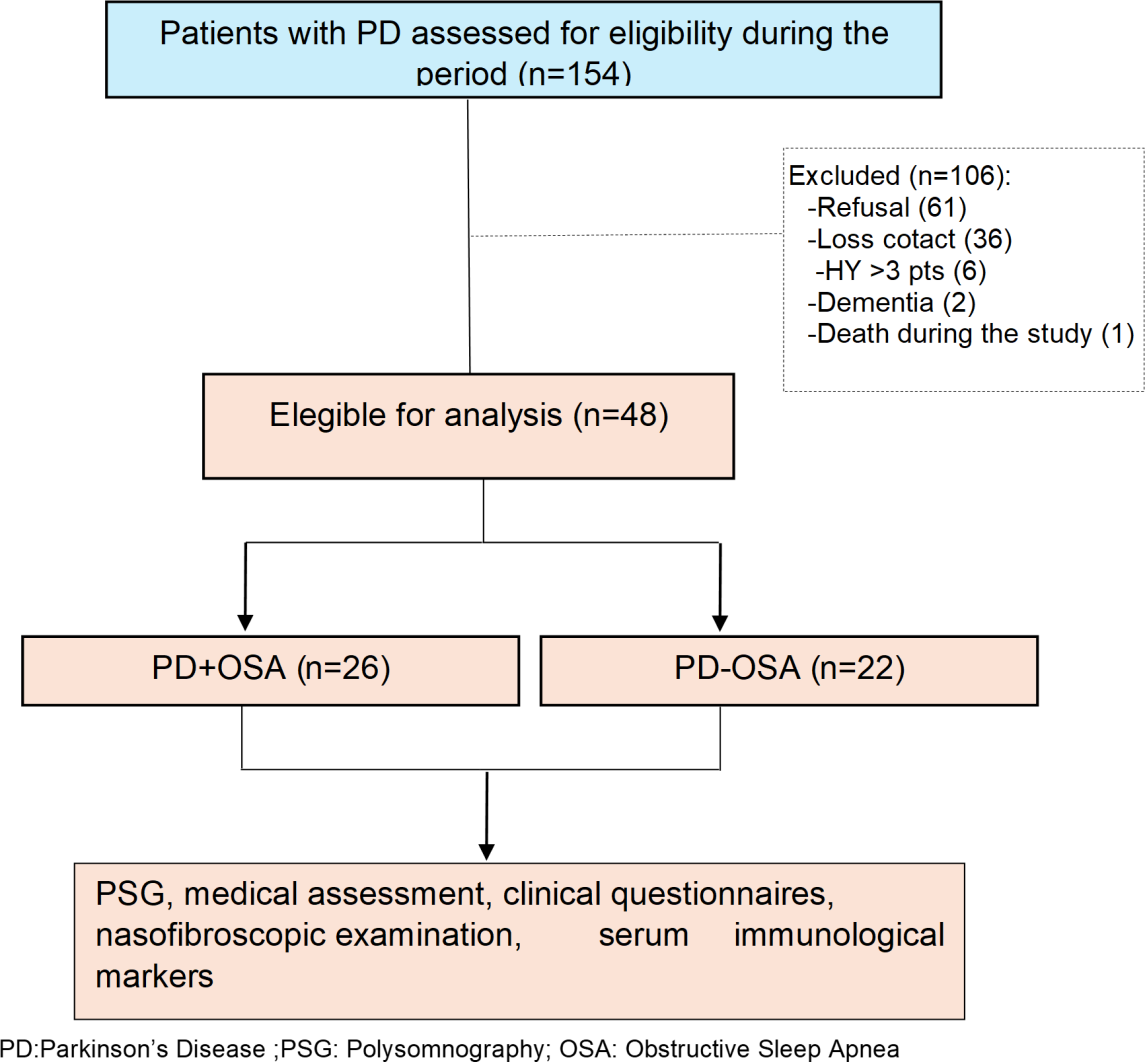
Flowchart of patient selection and study procedures.

A total of 154 patients with PD were assessed for eligibility, of whom 106 were excluded (refusal, loss of contact, Hoehn & Yahr stage >3, dementia, or death during follow-up). Forty-eight patients were eligible and included in the analysis, comprising 26 patients with PD and obstructive sleep apnea (PD+OSA) and 22 without obstructive sleep apnea (PD-OSA). All included participants underwent overnight PSG, medical assessment, clinical questionnaires, nasofibroscopic examination, and serum immunological marker evaluation (**Figure 1**).

Among the included patients, 26 were diagnosed with OSA, indicating a prevalence of 54.2%. Of these, two cases were classified as severe, nine as moderate, and fifteen as mild. In contrast, twenty-two patients were confirmed to have no OSA based on polysomnography (PSG). Poor sleep quality, defined by a PSQI score greater than 5, was observed in 87.5% of the sample (**Supplementary Tables 1**, **2**).

### 3.2 Clinical and sleep profile of PD patients with vs. without OSA

Patients with both PD and OSA (PD+OSA^+^ group) were significantly older (65.7 ± 11.2 years vs 57.9 ± 8.7 years, *p* = 0.01) and more likely to be married (*p* = 0,004) compared to those without OSA (PD+OSA^-^ group). There were no significant differences between groups in gender, education, BMI, neck circumference, disease duration, Hoehn & Yahr stage, Mallampati score, or Müller classification (all *p* > 0.05). Additional variables are detailed in **Table 1**.

**Table 1:** Clinical and sleep profile of PD patients with vs. without OSA.

Clinical and demographic characteristics	PD + OSA^+^ (n=26)	PD+ OSA^-^ (n=22)	P-value
Male sex, n(%)	15 (57.7%)	12 (54.5%)	0.83
Education level			0.69
Elementary school, n(%)	4 (15.4%)	1 (4.5%)	
High school, n(%)	15 (57.7%)	13 (59.1%)	
Incomplete higher education, n(%)	1 (3.8%)	2 (9.1%)	
Higher education,n(%)	5 (19.2%)	4 (18.2%)	
Postgraduate,n(%)	1 (3.8%)	2 (9.1%)	
A age (years), means ± SD	65.69 ± 11.25	57.95 (SD 8.72)	0.01*
Marital status			0.004*
Married, n(%)	19 (76.9%)	11 (50%)	
Divorced, n(%)	1 (3.8%)	4 (18.2%)	
Single, n(%)	1 (3.8%)	7 (31.8%)	
Widowed, n(%)	4 (15.4%)	0 (0%)	
MMSE,means ± SD	28.87 ± 1.00	28.98 ± 1.64)	0.148
Body Mass Index, means ± SD	27.45 ± 4.10	26.61 ± 3.02	0.54
Neck circumference (cm), means ± SD	37.50 ± 3.83	36.32 ± 5.15	0.6
Disease duration (years), means ± SD	8.15 ± 4.77	8.55 ± 6.89	0.89
Hoehn and Yahr			0.94
Stage 1, n(%)	5 (19.2%)	5 (22.7%)	
Stage 2, n(%)	8 (30.8%)	7 (31.8%)	
Stage 3,n(%)	13 (50%)	10 (45.5%)	
Sleep position			0.52
Supine, n(%)	7 (26.9%)	4 (18.2%)	
Lateral, n(%)	17 (65.4%)	14 (63.6%)	
Prone, n(%)	2 (7.7%)	4 (18.2%)	
Depression symptoms, n(%)	Yes 13 (50%)	Yes 13 (59.1%)	0.53
Anxiety symptoms, n(%)	Yes 17 (65.4%)	Yes 16 (72.7%)	0.58
ESS			0.86
No sleepiness, n(%)	10 (38.5%)	9 (40.9%)	
Sleepiness, n(%)	16 (61.5%)	13 (59.1%)	
Insomnia, n(%)	19 (76%)	12 (57,1%)	0,17
Mallampati score, n(%)	I/II 2 (15,4%)	I/II 3(27,3%)	0,36
	III/IV 11 (84,6%)	III/IV 8 (72,7%)	
Muller score, n(%)	I/II 4 (30,8%)	I/II 3 (27,3%)	1,00
	III/IV 9 (69,2%)	III/IV 8 (72,7%)	
STOP-BANG			0.79
Low risk, n(%)	3 (11.5%)	4 (18.2%)	
Intermediate risk, n(%)	12 (46.2%)	8 (36.4%)	
High risk, n(%)	11 (42.3%)	10 (45.5%)	
RLS, n(%)	Yes 11 (42.3%)	Yes 8 (36.4%)	0.68
RBDSQ			0.37
<5 pts, n(%)	14 (53.8%)	9 (40.9%)	
> 5pts, n(%)	12 (46.2%)	13 (59.1%)	
PDSS, means ± SD	101.62 (± 121.40)	76.64 ± 23.18	0.69
UPDRS I, means ± SD	18.04 ± 4.27	16.05 ± 15.13	0.07
UPDRS II, means ± SD	21.58 ± 7.16	16.82 ± 4.39	0.009*
UPDRS III, means ± SD	58.38 ± 23.55	45.32 ± 15.46	0.02*
UPDRS IV, means ± SD	4.27 ± 5.06	3.18 ± 4.15)	0.52
Last levodopa dose (mg), means ± SD	112.58 ± 70.15	132.27 ± 86.48	0.5
Presence of dyskinesias, n(%)	Yes 5 (19.2%)	Yes 6 (27.3%)	0.56

In the analysis (comparison) of categorical variables, we used the Chi-square (X²) test and the exact Chi-square test. For numerical variables, we applied the non-parametric Mann-Whitney test, except for the STOP-BANG, for which we applied for the non-parametric Kruskal-Wallis test. PD, Parkinson’s Disease; OSA, Obstructive Sleep Apnea; MMSE, Mini-Mental State Examination; ESS, Epworth Sleepiness Scale; RLS, Restless Legs Syndrome; RBD, REM Sleep Behavior Disorder; PDSS, Parkinson’s Disease Sleep Scale; UPDRS, Unified Parkinson’s Disease Rating Scale. Bold values indicate statistical significance.

* denotes statistical significance at p < 0.05.

Regarding UPDRS domains, the PD+OSA^+^ group showed significantly higher scores compared to the PD+OSA^-^ group in UPDRS-II (21.6 ± 7.2 vs. 16.8 ± 4.4; *p* = 0.009) and UPDRS-III (58.4 ± 23.6 vs. 45.3 ± 15.5; *p* = 0.02). There was a trend toward worse scores in UPDRS-I scores (18.04 ± 4.27 vs. 16.05 ± 5.14; *p* = 0.07), while no significant differences were observed in UPDRS-IV (**Table 2**).

**Table 2:** Polysomnographic characteristics of PD patients with and without OSA.

Variables	PD + OSA^+^ (N = 26)	PD+OSA^-^ (N = 22)	P-value
TST (minutes), means (SD)	334.88 (86.40)	409.66 (80.48)	0.008*
Sleep efficiency (%)	67.00 (15.55)	74.67 (13.44)	0.14
REM latency (minutes), means (SD)	166.06 (134.27)	190.55 (110.87)	0.39
N1 latency (minutes), means (SD)	13.79 (19.04)	12.80 (12.14)	0.75
N3 (minutes), means (SD)	80,4 (47,87)	90,48(38,48)	0,26
REM (minutes), means (SD)	30,88 (20,77)	48,44 (27,16)	**0,03**
WASO (minutes), means (SD)	146.59 (67.14)	117.74 (63.38)	0.19
RDI (events/hour), means (SD)	15.77 (11.34)	2.65 (1.44)	<0.001*
AHI (events/hour), means, (SD)	15.98 (11.20)	2.57 (1.50)	<0.001*
Desaturation Index, means (SD)	20.18 (16.28)	3.48 (3.68)	<0.001*
Minimum SpO2 time (%)	84.19 (7.54)	88.59 (6.30)	0.008*
Mean SpO2 (%), means (SD)	94.54 (1.88)	95.32 (1.59)	0.14
Heart rate (bpm), means (SD)	63.42 (9.47)	63.83 (9.39)	0.8
PLMi, means (SD)	6.84 (17.37)	6.16 (11.17)	0.01*
Snoring index, means (SD)	167.50 (247.50)	80.09 (184.73)	0.02*
Arousal index (events/hour), means (SD)	19.23 (10.95)	12.07 (12.61)	0.003*
Supine AHI, means (SD)	4.01 (7.28)	0.51 (1.01)	0.08
Non-supine AHI, means (SD)	4,20 (6.43)	0.62 (1.08)	**0.05**

For numerical variables, we applied the non-parametric Mann-Whitney test.

AHI, Apnea-Hypopnea Index; PLMi, Period Leg Movements index; OSA, Obstructive Sleep Apnea; REM, Rapid Eye Movement Sleep; RDI, Respiratory Disturbance Index; SD, Standard Deviation; TST, Total Sleep Time; WASO, Awake After Sleep. Bold values indicate statistical significance.

* denotes statistical significance at p < 0.05.

Polysomnographic analysis (**Table 2**) revealed that the PD+OSA+ group had significantly shorter total sleep time (334.9 ± 86.4 vs. 409.7 ± 80.5 minutes; *p* = 0.008), reduced REM sleep duration (30.9 ± 20.8 vs. 48.4 ± 27.2 minutes; *p* = 0.03), and a higher arousal index (19.2 ± 10.9 vs. 12.1 ± 12.6; *p* = 0.003) compared to the PD+OSA- group. Respiratory indices, including RDI (15.8 ± 11.3 vs. 2.7 ± 1.4; *p* < 0.001), AHI (15.7 ± 11.3 vs. 2.6 ± 1.5; *p* < 0.001), desaturation index (20.2 ± 16.3 vs. 3.5 ± 3.7; *p* < 0.001), and minimum oxygen saturation (84.2 ± 7.5% vs. 88.6 ± 6.3%; *p* = 0.008), were also significantly higher in the PD+OSA+ group. Additionally, the periodic limb movement index (PLMI; *p* = 0.02) and non-positional apnea (*p* = 0.05) were elevated in the PD+OSA+ group. No other significant differences were observed between groups (**Table 2**).

In the multivariate binary logistic regression, the final model identified age, desaturation index, and snore index as significant predictors of OSA. Each additional year of age increased the odds of OSA by 12% (OR = 1.12; 95% CI: 1.01–1.25; *p* = 0.03). Each unit increase in the desaturation index raised the odds by 24% (OR = 1.24; 95% CI: 1.07–1.44; *p* = 0.004). Each unit increase in the snore index was associated with a small but statistically significant increase in the odds of OSA (OR = 1.005; 95% CI: 1.000–1.009; *p* = 0.03).

### 3.3 OSA is associated with immune reorganization in PD

**Figure 2:**
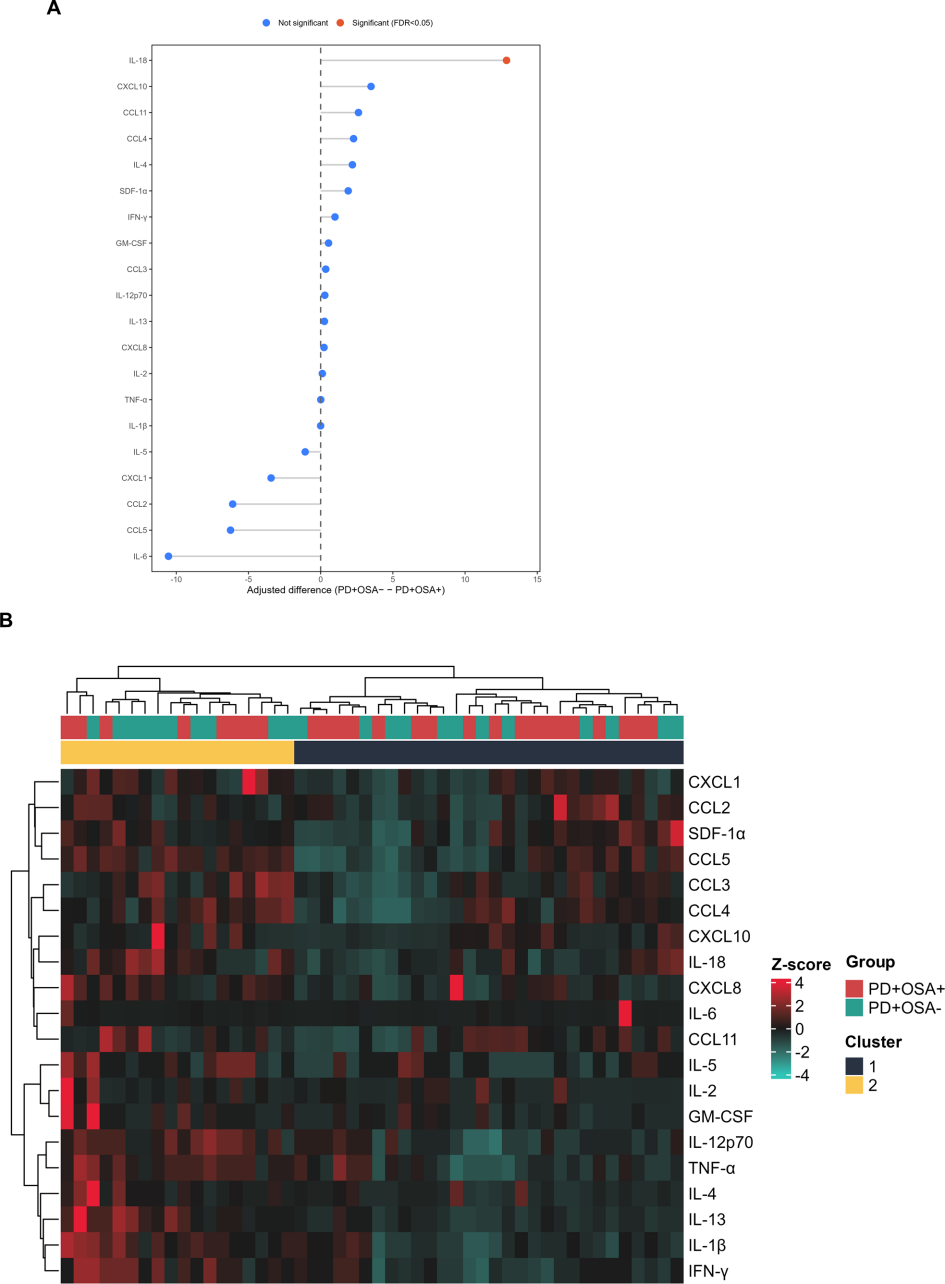
Group-specific cytokine signatures integrating ANCOVA-adjusted differences and clustering by expression patterns. **(A)** Forest plot of estimated adjusted differences in cytokine concentrations between PD+OSA− and PD+OSA+ groups, derived from models including age and sex as covariates. Each point represents the coefficient for the group effect (PD+OSA− − PD+OSA+), with horizontal bars showing the 95% confidence interval. Positive values indicate higher adjusted levels in PD+OSA−, and negative values indicate higher levels in PD+OSA+. Colors denote statistical significance after FDR correction (orange = significant, FDR < 0.05; blue = not significant) **(B)** Heatmap of standardized cytokine concentrations (z-scores), clustered by Euclidean distance and Ward.D2 linkage for both rows and columns. Annotation bars indicate diagnostic group and cluster membership. Color scale centered at zero. Heatmap de z-scores (padronização por citocina), distância Euclidiana e ligação Ward.D2 para linhas e colunas, com barras de anotação para grupo e cluster. Escala de cor centrada em 0.

Univariate comparison of plasma cytokine levels between the PD+OSA^−^ and PD+OSA^+^ groups revealed a statistically significant difference for a single cytokine, IL-18, which was elevated in the PD+OSA− group (*p* = 0.003; **Figure 2A**). This suggests that, individually, most cytokines did not discriminate between groups. However, multivariate analysis using standardized z-scores revealed the underlying immune substructures in both groups. The heatmap (**Figure 2B**) identified two major sample clusters, each defined by distinct combinatorial cytokine expression patterns.

One cluster, predominantly composed of PD+OSA^+^ individuals, showed high and coordinated expression of leukocyte-recruiting chemokines — including CXCL1 (GRO-α), CCL2 (MCP-1), CCL3 (MIP-1α), CCL4 (MIP-1β), CCL5 (RANTES), CXCL8 (IL-8), and SDF-1α — along with IL-6. This chemokine-dominant profile is consistent with an inflammatory milieu associated with endothelial and innate immune signaling, and may reflect enhanced leukocyte trafficking, including monocyte, neutrophil and T cell-related chemotactic processes (e.g., via CXCR3- associated pathways), as reported in systemic inflammatory conditions linked to intermittent hypoxia.

Absolute quantification showed that SDF-1α was the most abundant analyte across the entire cohort, whereas IL-18 remained the only cytokine to distinguish groups in univariate comparisons (**Supplementary Figure 1**). These findings indicate that, in our study population, differences between OSA+ and OSA− patients are more apparent when considering multivariate immune signatures rather than individual cytokine levels.

### 3.4 OSA is associated with systemic differences in inflammatory network organization in PD

**Figure 3:**
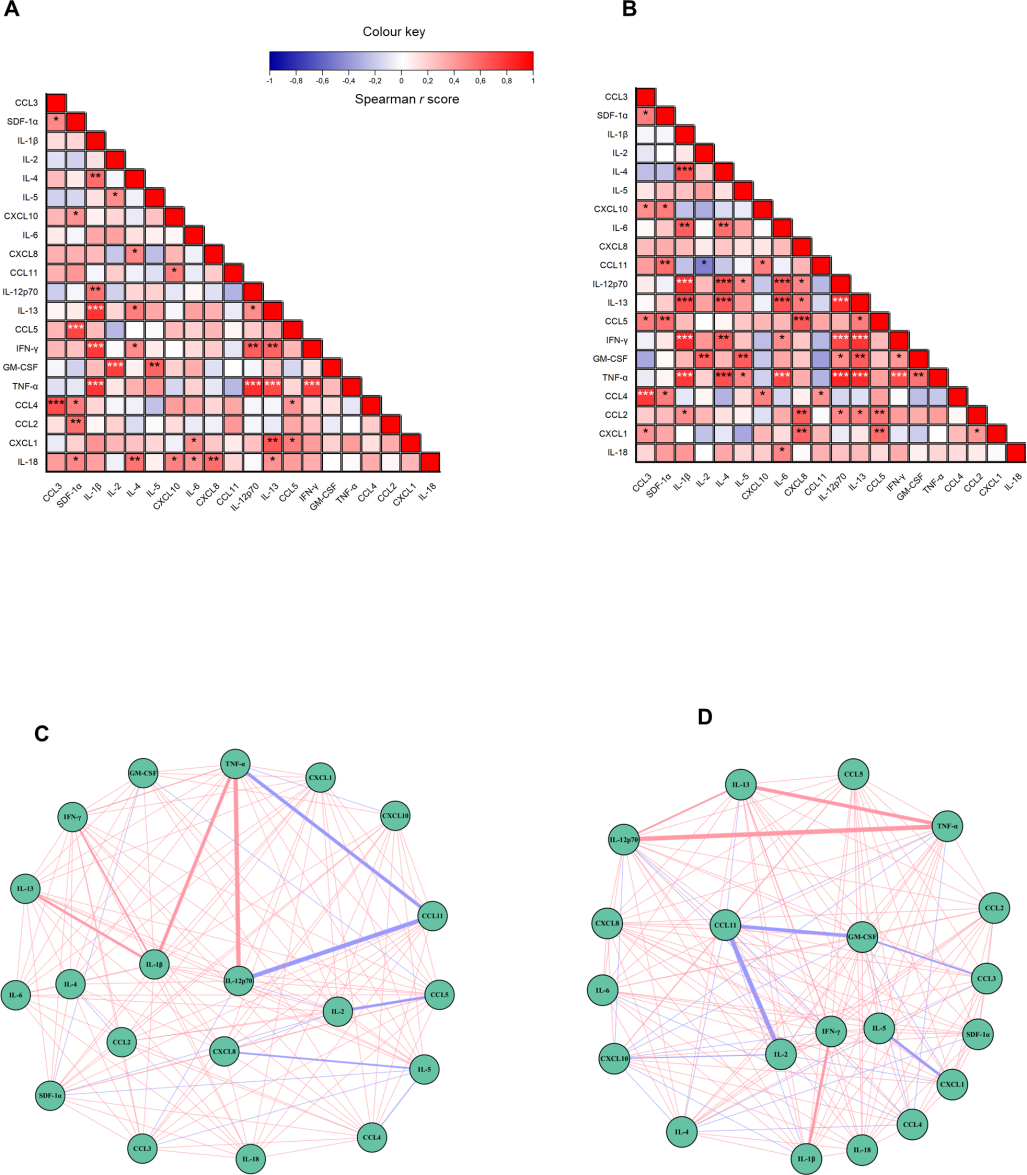
Differential cytokine correlation networks in PD patients with (PD+OSA+) and without (PD+OSA-) comorbid OSA. **(A, B)** Spearman correlation matrices of cytokine pairs in PD+OSA– **(A)** and PD+OSA+ **(B)**. Color scale indicates correlation strength (red = positive, blue = negative); numerical values denote correlation coefficients. Statistical significance is marked by asterisks (*p < 0.05; **p < 0.01; ***p < 0.001). **(C, D)** Undirected correlation networks for PD+OSA– **(C)** and PD+OSA+ **(D)**, built from the same correlation matrices. Node size reflects degree, edge color indicates correlation sign (red = positive, blue = negative), and edge thickness is proportional to correlation magnitude.

Spearman correlation analysis revealed clear differences in cytokine–cytokine interaction patterns between the PD+OSA^−^ and PD+OSA^+^ groups. In the PD + OSA − group (**Figure 3A**), 24 strong positive correlations were identified (ρ ≥ 0.60; p < 0.05). These appeared as localized blocks of associations involving subsets of cytokines, such as IL-18, IL-1β, and TNF-α, and chemokines, such as CCL2 and CXCL10, with minimal interconnection across modules. This pattern reflects a compartmentalized network of inflammatory mediators.

The corresponding network graphs (**Figure 3C**) mirror this organization, displaying a lower edge density, fragmented structure, and a few negative (blue) connections. Only some cytokines, such as IL-18 and CCL2, appeared centrally positioned, but there were no extensive bridging nodes. This architecture suggests limited systemic coupling between the inflammatory pathways in PD+OSA−.

In contrast, the PD+OSA^+^ group exhibited a markedly broader and more integrated interactive landscape. The correlation matrix (**Figure 3B**) showed 44 strong positive correlations spanning both innate and adaptive mediators, including IL-1β, IL-6, TNF-α, IFN-γ, IL-12p70, IL-4, IL-5, IL-13, CCL2, CCL4, CCL5, CXCL1, CXCL8, and CXCL10. This integration was evident in the PD+OSA+ network (**Figure 3D**), which displays a dense and highly connected topology. A visually central inflammatory core emerges, with IL-6, IL-1β, TNF-α, CXCL8, CCL2, and CXCL1 appearing as key nodes linked to Th2 cytokines (IL-4, IL-5, and IL-13) through chemokines. Thicker red edges indicate higher |ρ| values, whereas blue edges remain rare and peripheral, underscoring the predominance of positive, coordinated signaling. Comparison of PD+OSA^−^ (**Figures 3A, C**) and PD+OSA^+^ (**Figures 3B, D**) revealed a clear shift from a fragmented, low-connectivity network to a densely integrated inflammatory system driven by coordinated chemokine and cytokine activation.

**Figure 4:**
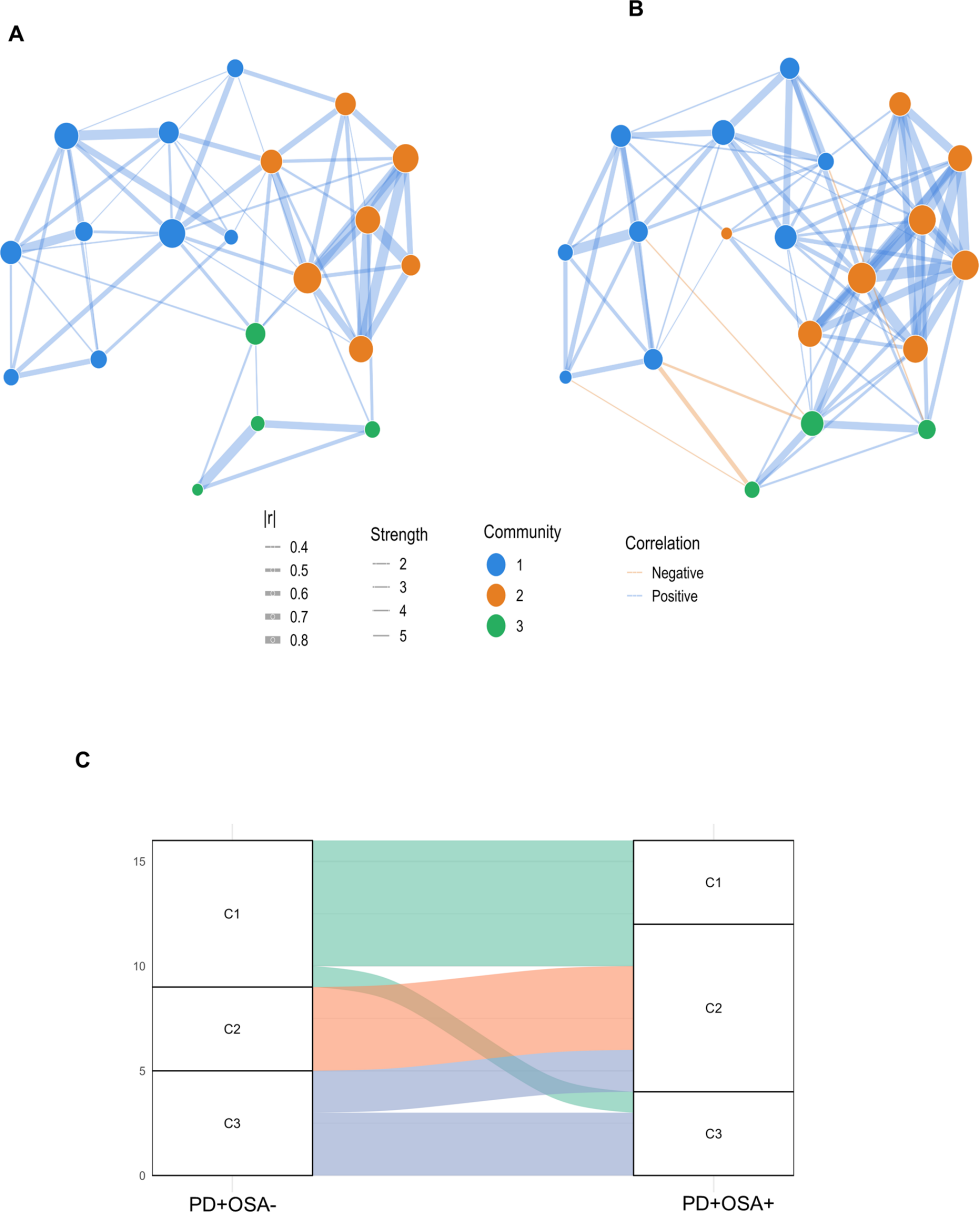
Cytokine Louvain communities and intergroup reorganization. **(A, B)** Undirected correlation networks for PD+OSA– **(A)** and PD+OSA+ **(B)**, constructed from cytokine pairs with |ρ| ≥ 0.40 (Spearman). Edge width is proportional to correlation magnitude, with blue edges denoting positive and orange edges negative correlations. Node color represents community assignment identified by the Louvain algorithm. **(C)** Sankey diagram depicting the redistribution of cytokines between Louvain communities in PD+OSA– and PD+OSA+. The flows highlight the reorganization of cytokine modules toward more integrated community structures in PD+OSA+.

Functional analysis suggests that OSA is associated with coordinated activation of major inflammatory pathways (e.g., IL-1/IL-6/TNF axis and neutrophil/monocyte chemotactic signaling), with enhanced leukocyte recruitment and amplified systemic immune responses. This widespread immune reorganization was confirmed using the Louvain community detection algorithm. In PD + OSA−individuals, immune networks formed smaller, sparsely connected communities (C1–C3; **Figure 4A**). In contrast, the PD+OSA+ group showed the emergence of larger and more cohesive network modules (**Figure 4B**), characterized by stronger intermodular connectivity (ρ ≥ 0.7). Further evidence from the Sankey plot analysis (**Figure 4C**) revealed that cytokines initially located at the periphery in the PD+OSA^−^ group (communities 1 and 3) shifted toward central inflammatory modules in PD+OSA^+^ individuals (notably, communities 2 and 3). This pattern suggests that OSA is associated with more centralized and intensified immune signaling.

### 3.5 OSA intensity drives a dominant inflammatory axis

**Figure 5:**
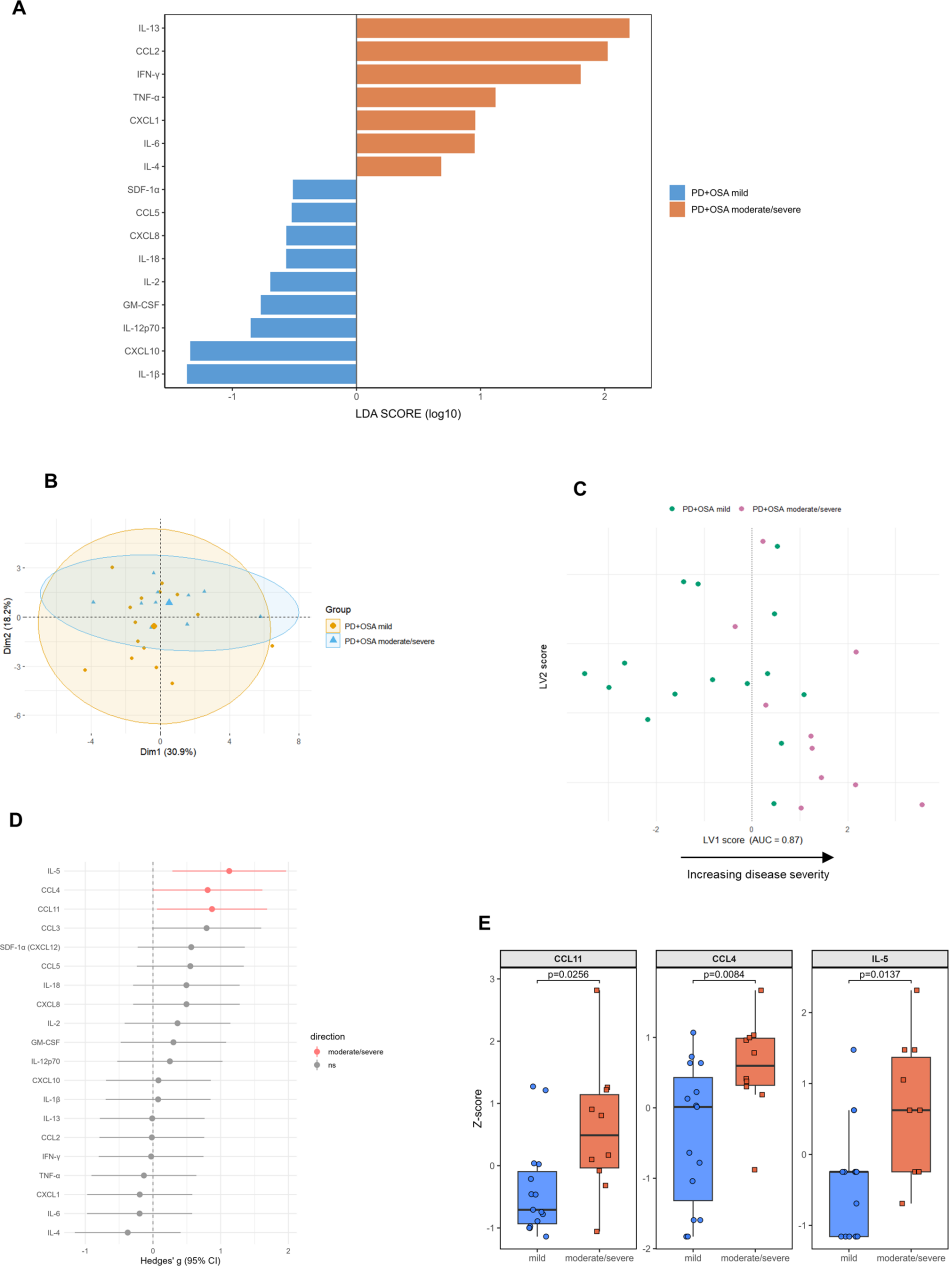
Cytokine discrimination according to OSA severity in PD patients. **(A)** Linear Discriminant Analysis (LDA) showing cytokines associated with mild (blue) and moderate/severe (orange) OSA subgroups. **(B)** Principal Component Analysis (PCA) of cytokine profiles, illustrating partial separation of mild and moderate/severe groups with some overlap. **(C)** Latent Variable 1 (LV1) scores from multivariate modeling, with AUC = 0.87 (leave-one-out cross-validation) for classifying mild vs moderate/severe subgroups. Patients with mild OSA cluster toward negative LV1 values, while moderate/severe cases cluster toward positive LV1 values. The arrow indicates the direction of increasing disease severity along the latent axis. **(D)** Forest plot of standardized between-group differences (Hedges’ g) for each cytokine; points show the effect size (moderate/severe – mild) and horizontal bars the 95% CI, ordered by |g|. Red points denote cytokines higher in the moderate/severe group with p < 0.05 (two-tailed Welch’s); grey indicates non-significant results. **(E)** Boxplots of IL-5, CCL4, and CCL11 showing higher levels in moderate/severe patients (two-tailed Welch’s t-test, FDR FDR<0.05); For comparability across analytes, axes are shown as z-scores, although statistical tests were performed on the original measurements.

Stratification of PD+OSA^+^ patients by OSA severity (mild vs. moderate/severe) revealed distinct immune signatures. Linear Discriminant Analysis (LDA; **Figure 5A**) effectively separated the subgroups, with elevated scores for IL-13, CCL2, IFN-γ, TNF-α, CXCL1, IL-6, and IL-4 in the moderate/severe group. This profile indicates a mixed inflammatory state involving Th1 signaling, innate myeloid activation, and Th2-associated tissue responses. Conversely, the mild OSA group showed relative enrichment of IL-1β, CXCL10, IL-12p70, GM-CSF, IL-2, IL-18, CXCL8, CCL5, and SDF-1α — suggesting a pattern associated with early phase innate activation, antigen presentation, angiogenesis, and chemotaxis.

Principal Component Analysis (PCA; **Figure 5B**) further supported group separability, with overlapping yet distinct clustering tendencies and 95% confidence ellipses indicating intragroup variability. Latent variable analysis (LV1; **Figure 5C**) provided robust discrimination between severity groups (AUC = 0.87, LOOCV), with positive scores predominantly in the moderate/severe group and negative scores predominantly in the mild group, reflecting a gradient of inflammatory consolidation.

To link multivariate findings to individual markers, we performed univariate analyzes. A forest plot of standardized mean differences (Hedges’ g; **Figure 5D**) summarized effect sizes across all analytes. IL-5 (p = 0.0137), CCL4 (p = 0.0084), and CCL11 (p = 0.0256) showed the strongest positive effects with 95% confidence intervals not crossing zero, indicating significant elevations in the moderate/severe group (two-tailed Welch’s t-test, FDR < 0.05; **Figure 5E**). Other markers generally showed similar trends to the multivariate analysis, but their confidence intervals overlapped zero, indicating non-significant differences.

The data show that OSA severity in patients with PD influences the organization of inflammatory responses, with moderate/severe cases displaying more coordinated and clustered biomarker profiles, while mild cases exhibit a more heterogeneous and compartmentalized pattern.

## 4 Discussion

To date, few studies have examined the impact of OSA on PD using full PSG alongside inflammatory markers (5). Our study found a high prevalence of OSA (54.2%) among patients with PD. Those with PD and OSA were older, had poorer quality of life, greater motor impairment, and more severe non-motor symptoms compared to those without OSA. PSG data showed that patients with OSA had shorter total sleep time, higher arousal index, higher desaturation index, reduced REM sleep, and more non-positional apneas, which is consistent with the known pathophysiology of OSA and its impact on PD outcomes (8, 10).

OSA is increasingly recognized as a clinically relevant comorbidity in PD, potentially linked by convergent neuroinflammatory mechanisms. Experimental evidence indicates that hypoxia drives mitochondrial dysfunction, microglial activation and oligodendrocyte injury, resulting in demyelination and neuroinflammation (34, 35). This chronic inflammatory state may amplify degenerative cascades through hypoxia-inducible factors (HIFs), creating a “double-hit” — where both neuroinflammation and impaired glymphatic clearance act together to accelerate PD progression mechanism that worsens neuroinflammation, impairs glymphatic clearance, and accelerates PD progression (17, 36–38).

In our cohort, OSA was associated with differences in cytokine profiles in PD, with distinct immune patterns observed between groups. Although IL-18 levels were unexpectedly higher in the PD + OSA^-^ group, multivariate analyzes showed that OSA was associated with a broader immune reorganization rather than a single cytokine change. The PD + OSA^+^ group displayed a chemokine-enriched signature indicative of endothelial activation and leukocyte recruitment, alongside interconnected IL-1/IL-6/TNF networks. Stratification revealed that mild OSA showed a pattern of innate immune activation, whereas moderate-to-severe OSA showed a pattern of mixed Th1/Th2 adaptive response.

The paradoxically higher IL-18 in PD + OSA^-^ may reflect compensatory or exhaustion mechanisms, where chronic PD inflammation sustains inflammasome activity while hypoxia in OSA induces feedback desensitization. Possible mechanisms include IL-18 binding protein upregulation (39), inflammasome desensitization under chronic hypoxia (40), or miR-22–mediated suppression of NLRP3/caspase-1 (41). Although untested here, these hypotheses warrant further study. Overall, OSA is associated with differences in inflammatory network organization in PD, with distinct patterns observed between groups. To our knowledge, this immune pattern has not been previously described in PD with comorbid OSA.

OSA is characterized by a state of chronic low-grade inflammation driven primarily by intermittent hypoxia and sleep fragmentation (42, 43). These pathophysiological stressors lead to increased circulating levels of pro-inflammatory cytokines, including IL-6, TNF-α, IL-1β, and IL-18, as well as chemokines such as CCL2 and IL-8, which collectively promote oxidative stress, endothelial dysfunction, and systemic inflammatory activation (42). Sustained exposure to intermittent hypoxia further amplifies inflammatory signaling through activation of the NLRP3 inflammasome, thereby maintaining IL-1β and IL-18 release and perpetuating immune dysregulation (41). Such immune alterations contribute to metabolic and vascular impairment and may represent a mechanistic link between OSA and neurodegenerative processes.

In this context, early identification and treatment of OSA may attenuate neuroinflammatory burden, particularly through modulation of key inflammatory mediators such as IL-6, CCL2, and IL-18 (42, 44). Zhu et al. (45) demonstrated that CPAP therapy significantly reduced IL-6, TNF-α, and IL-8 levels, reinforcing a causal relationship between hypoxia and cytokine activation. In line with these findings, Kaminska et al. (5) showed that elevated IL-6 levels were associated with both OSA severity and motor impairment in PD. Notably, a recent nationwide cohort study further demonstrated that OSA independently increases the risk of incident PD (18). Early CPAP treatment substantially attenuates this risk, highlighting OSA as a modifiable midlife factor in the neurodegenerative cascade (17).

In our cohort, PD+OSA^+^ patients showed fragmented sleep, hypoxemia, and increased limb movements, correlating with poorer quality of life and higher UPDRS scores. These findings support the role of OSA in enhancing neuroinflammatory vulnerability in PD, possibly related to intermittent hypoxia and sleep disruption. Our results highlight the potential benefit of early OSA screening and treatment to help mitigate PD progression.

Future directions include conducting more clinical trials using CPAP therapy to determine whether treating OSA can reduce the observed inflammatory network, potentially slow PD progression, and improve cognitive outcomes. This represents a crucial gap in cross-sectional studies like ours. Innovative approaches might incorporate neuroimaging to explore glymphatic dysfunction or genetic studies examining polymorphisms in inflammatory genes such as *TNF-α* in PD+OSA cohorts. These longitudinal strategies would help clarify causal relationships and evaluate the impact of early interventions, thereby building on our current findings related to persistent inflammation.

Our study had some limitations that should be carefully considered, including its cross-sectional design, which precludes causal inference; the relatively small sample size, which may have limited statistical power in some analysis; potential selection bias toward patients with more prominent sleep complaints; and the absence of a non-PD control group, which further restricts the generalizability of our findings. In addition, the chemokine and cytokine panel assessed was limited and selected to address specific hypothesis-driven questions. Given the pleiotropic nature of many of the immune mediators assessed, the present findings should be interpreted as evidence of systemic immune–inflammatory remodeling rather than as proof of activation of any single molecular pathway. Nevertheless, unlike previous studies, we investigated potential pathophysiological mechanisms involved in OSA and interpreted the observed associations within a plausible biological framework. We also enhanced diagnostic accuracy through physician-conducted assessments and laboratory-based polysomnography.

## 5 Conclusion

OSA is prevalent in PD, affecting more than half of the patients, and it is linked to poorer sleep quality, reduced quality of life, and worsening motor function. This study further demonstrates that OSA is associated with a sustained proinflammatory state in PD, particularly in moderate-to-severe cases, characterized by a complex cytokine and chemokine network. This inflammatory profile may be associated with disease progression, highlighting the importance of early OSA detection and treatment as a key part of comprehensive PD care.

## Data Availability

The original contributions presented in the study are included in the article/**Supplementary Material**. Further inquiries can be directed to the corresponding authors.

## Supplementary material

Supplementary Figure 1 Absolute cytokine distributions by group. Boxplots (median, interquartile range, individual data points) for all cytokines. SDF-1α showed the highest absolute concentrations across the cohort. IL-18 confirmed group differences between PD+OSA– and PD+OSA+ in univariate analysis. Group comparisons were performed using two-tailed Mann–Whitney tests with p-value correction for multiple testing.
