# Supplementary material for: Obstructive sleep apnea is linked to inflammatory changes and motor impairment in Parkinson’s disease
Source: Front Immunol. 2026 May 18;17:1808550. doi: 10.3389/fimmu.2026.1808550 (PMC13223042; doi:10.3389/fimmu.2026.1808550)
Supplement: Supplementary file 1 [file Table1.docx]

| **Supplementary table 1 - Baseline clinical characteristics”** | |
| --- | --- |
| **Variables** | **N=48** |
| **Age (years), mean (SD)** | 62,1 (10,79) |
| **Gender, male (%)** | 27(52,2) |
| **BMI, mean (SD)** | 27,0 (3,63) |
| **CC, mean (SD)** | 36,9(4,4) |
| **Years with PD, mean (SD)** | 8,3 (5,7) |
| **Hoehn-Yahr, mean (SD)** | 2,2 (0,79) |
| **ESS, mean (SD)** | 12,5 (7,01) |
| **UPDRS-I, mean (SD)** | 17,1 (4,73) |
| **UPDRS-II, mean (SD)** | 19,3 (6,45) |
| **UPDRS-III, mean (SD** | 52,3 (21,10) |
| **PDSS, mean (SD)** | 90,1 (90,76) |
| **PSQi, mean (SD)** | 8,8 (4,14) |
| **MMSE, mean (SD)** | 28,5 (1,35) |

BMI: body mass index; CC=Cervical circumference; ESS= Epworth Sleep Scale; PD = Parkinson Disease; UPDRS; *Unified Parkinson´s Disease Rating Scale; N=* number of valid records for that variable; PDSS= *Parkinson´s Disease Sleep Scale;* PSQI: Pittsburgh Sleep Quality Index; MEEM = Mini Mental State Examination.
