# Supplementary material for: Obstructive sleep apnea is linked to inflammatory changes and motor impairment in Parkinson’s disease
Source: Front Immunol. 2026 May 18;17:1808550. doi: 10.3389/fimmu.2026.1808550 (PMC13223042; doi:10.3389/fimmu.2026.1808550)
Supplement: Supplementary file 2 [file Table2.docx]

**Supplementary table. 2–Sleep quality and sleep disorders**

| **Variables** | **N (%)** |
| --- | --- |
| **PSQi** | |
| Poor sleep quality | 42 (87.5) |
| Good sleep quality | 6 (12.5) |
| **Insomnia** | 31 (64.5) |
| **EES** | |
| Somnolance | 29 (60.4) |
| No Somnolance | 19 (39.6) |
| **OSA** | 26 (54.2) |
| **STOP-BANG** | |
| High risk | 21 (43.8) |
| Intermdiate risk | 20 (41.7) |
| Low risk | 7 (14.6) |
| **RLS** | |
| No | 29 (60.4) |
| Yes | 19 (39.6) |
| **RBD** | 14 (29,1) |

N = Number of valid records for that variables; EDS: Excessive Diurnal Somnolence; PSQi= *Pittsburgh Sleep Questionnare índex; RLS* = Restless Legs Syndrome; RBD = Rem Sleep Behaviour Disorder; EES= Epworth Sleep Scale.
