# Supplementary figures and images for: Obstructive sleep apnea is linked to inflammatory changes and motor impairment in Parkinson’s disease
Source: Front Immunol. 2026 May 18;17:1808550. doi: 10.3389/fimmu.2026.1808550 (PMC13223042; doi:10.3389/fimmu.2026.1808550)

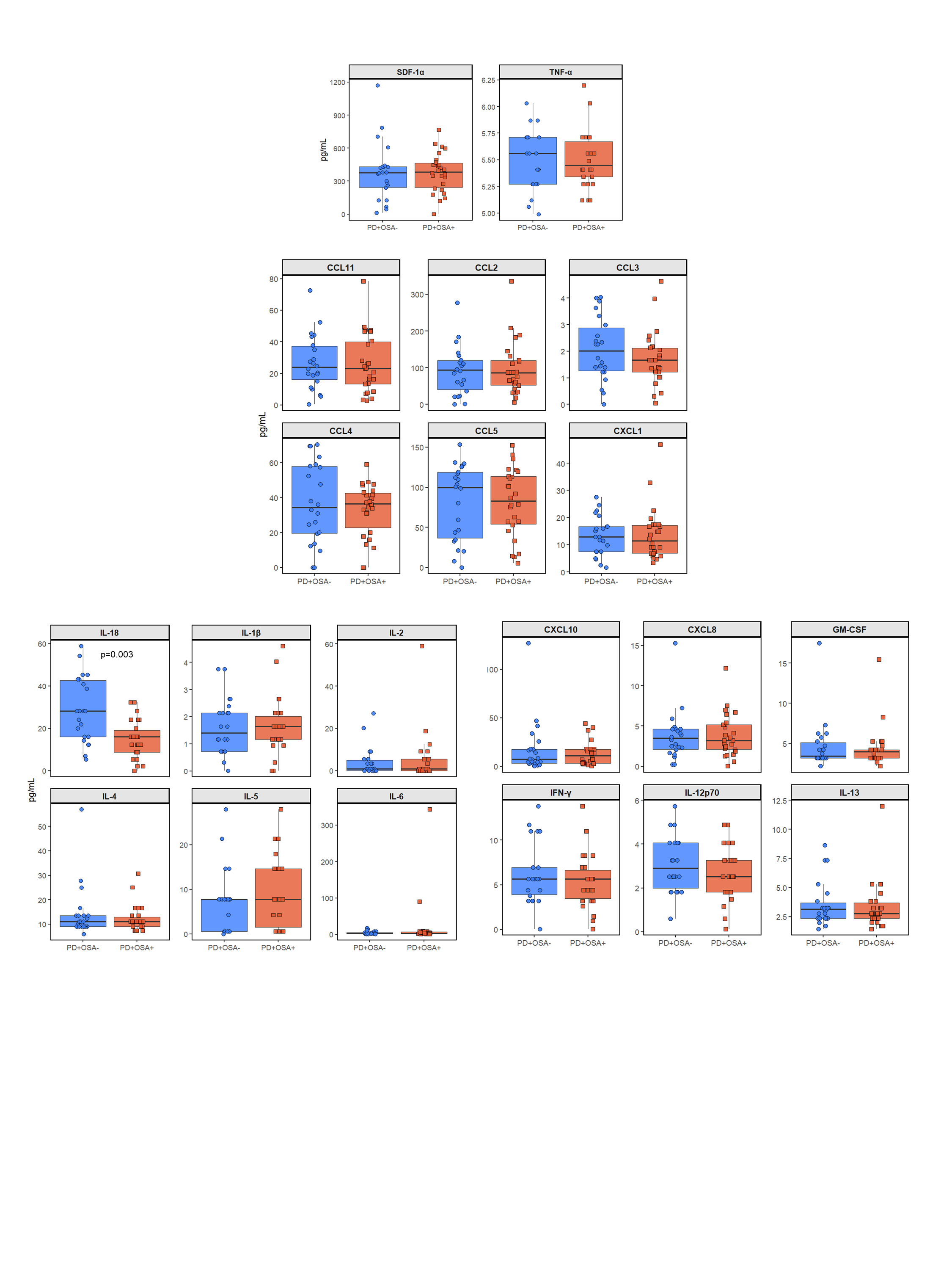

Supplement: Supplementary Figure 1 — Absolute cytokine distributions by group. Boxplots (median, interquartile range, individual data points) for all cytokines. SDF-1α showed the highest absolute concentrations across the cohort. IL-18 confirmed group differences between PD+OSA– and PD+OSA+ in univariate analysis. Group comparisons were performed using two-tailed Mann–Whitney tests with p-value correction for multiple testing. [file Image1.tif]
